# Accelerated iTBS-Induced changes in resting-state functional connectivity correspond with cognitive improvement in amnestic MCI

**DOI:** 10.1016/j.brs.2025.04.012

**Published:** 2025-04-17

**Authors:** Stephanie Aghamoosa, Sara A. Nolin, Andrew A. Chen, Kevin A. Caulfield, James Lopez, Katrina Rbeiz, Holly H. Fleischmann, Olivia Horn, Katrina Madden, Michael Antonucci, Gonzalo Revuelta, Lisa M. McTeague, Andreana Benitez

**Affiliations:** a Department of Health Sciences and Research, Medical University of South Carolina, Charleston, SC, USA; b Center for Biomedical Imaging, Medical University of South Carolina, Charleston, SC, USA; c Department of Neurology, Medical University of South Carolina, Charleston SC, USA; d Department of Public Health Sciences, Medical University of South Carolina, Charleston, SC, USA; e Department of Psychiatry and Behavioral Sciences, Medical University of South Carolina, Charleston, SC, USA; f Department of Radiological Science, Medical University of South Carolina, Charleston, SC, USA; g Ralph H. Johnson VA Health Care System, Charleston, SC, USA

**Keywords:** Intermittent theta burst, Repetitive transcranial magnetic stimulation, Mild cognitive impairment, Alzheimer’s disease, Functional connectivity, Cognition

## Abstract

**Background:**

Published results of our Phase I safety and feasibility trial of accelerated intermittent theta burst stimulation (a-iTBS) in mild cognitive impairment (MCI) due to Alzheimer’s disease showed a large effect-size improvement in cognition.

**Objective:**

Further demonstrate target engagement by identifying whether changes in local and network-level functional connectivity relate to the observed cognitive improvement.

**Methods:**

Eighteen patients with MCI received 3-day a-iTBS (8 sessions/day) to the left dorsolateral prefrontal cortex at Beam F3 (14,400 total pulses) and completed MRI and cognitive testing at pre- and post-treatment. Based on electric field models, we selected 3 stimulated target regions of interest (ROIs) which belonged to the frontoparietal (FPN), default mode (DMN), and ventral attention (VAT) networks (3 target networks). Metrics of resting-state functional connectivity were computed at the ROI level (within-network degree: number of connections) and network level (segregation: strength of connectivity within-network relative to other networks). We correlated changes in cognition and connectivity of the target ROIs and networks; off-target ROI (primary visual) and networks served as negative controls.

**Results:**

Improvements in cognition were associated with connectivity changes in the target ROIs and networks, but not in off-target negative controls. Positive associations were observed for degree of the l-DMN and segregation of target networks overall, with significant effects for DMN and VAT.

**Conclusion:**

Cognitive improvement following a-iTBS in MCI may be attributable to local and network-level reconfigurations in functional connectivity. These findings will inform larger trials designed to further evaluate the neural mechanisms of a-iTBS for cognition in MCI.

## 1. Introduction

Accumulating evidence suggests that repetitive transcranial stimulation (rTMS) to left dorsolateral prefrontal cortex (l-dlPFC) improves cognition in people with neurodegenerative conditions, such as mild cognitive impairment (MCI) and dementia due to Alzheimer’s disease (AD) [1–3]. Application of rTMS earlier in the course of AD—during the MCI stage in which individuals exhibit cognitive deficits but remain functionally independent—is particularly promising given the potential to slow progression to dementia. Accelerated rTMS protocols, such as accelerated intermittent theta burst stimulation (a-iTBS), offer improved treatment feasibility for cognitively impaired older adults as they condense the same number of conventional rTMS treatments (i.e., one session daily for 4–6 weeks) into as few as 3 multi-session days. We recently conducted an open-label Phase I trial of 3-day a-iTBS in people with MCI due to AD that found treatment to be safe, feasible, and highly acceptable while producing a significant and large effect size improvement in fluid cognition [4]. The present study further substantiates a-iTBS as a treatment for MCI by assessing target engagement (i.e., demonstrating that a treatment affects an expected biological and/or behavioral outcome), evaluating the correspondence between change in cognition and brain functional connectivity following a-iTBS treatment.

Stimulation of superficial cortex with rTMS induces changes in brain activity both locally and across distally connected brain regions that comprise the large-scale functional networks of the brain [5,6]. The l-dlPFC is a common target of rTMS for MCI given that these brain regions play integral roles in cognitive control (i.e., executive function [7]), which is commonly deficient in MCI [8] and contributes to impairments in memory [9–11]. Regions of the l-dlPFC serve as hubs of several of the brain’s association networks (i.e., networks supporting higher-order cognitive processes), including the frontoparietal network (FPN), default mode network (DMN), and ventral attention network (VAT). These association networks are particularly relevant for MCI as they demonstrate alterations in function over the course of AD [12] that are associated with increasing cognitive deficits [13]. Individuals with MCI exhibit abnormal patterns of network interactions (i.e., connectivity within and between them) such that the networks become less separable (i.e., segregated) from each other [13,14]. Thus, the central premise of excitatory rTMS to l-dlPFC as a treatment for MCI is that it will increase activity in l-dlPFC regions and correct dysfunctional network interactions, thereby improving executive function and supporting memory. Meta-analytic evidence supports this notion at the behavioral level, showing that rTMS to l-dlPFC improves executive function, memory, and global cognition in MCI [2,3]. However, there is currently limited investigation of the functional neural mechanisms of these effects.

The goal of this study was to build upon the preliminary evidence of improved cognition (i.e., behavioral change) from our Phase I trial [4] by investigating whether this change relates to functional brain changes, thus further substantiating target engagement and identifying the functional neural mechanisms of a-iTBS for MCI. We hypothesized that a-iTBS would have effects on brain functional connectivity at both the local level (i.e., regions under the stimulation target) and network level (i.e., among regions of the stimulated target networks) that correspond to cognitive improvement. To test this, we used functional connectivity metrics expected to be sensitive to a-iTBS effects at these two different scales. First, we evaluated local a-iTBS effects using within-network degree, which measures a region’s number of functional connections to other regions in its network (with higher degree indicating a region is more “hub-like”). Second, we measured network-level a-iTBS effects using segregation, a metric of functional interactions within relative to between networks (with higher segregation indicating a “healthy” network organization that shows stronger connectivity within itself than to others) [15]. We hypothesized that cognitive improvement would correlate with increases in within-network degree of target regions and segregation of target networks, but not with negative-control (i.e., off-target) regions and networks.

## 2. Materials and methods

### 2.1. Participants

Participants with MCI were referred to this study from outpatient neurology clinics. As described previously [4], participants were between ages 60–85 years, had been diagnosed with MCI due to possible AD (per NIA-AA criteria [16]) within the past two years, demonstrated amnestic impairment on clinical neuropsychological assessment per actuarial criteria that improve diagnostic specificity and stability [17, 18], and spoke English as their first and/or primary language. Twenty-four participants were enrolled, 22 initiated treatment, and 18 had complete and usable neurocognitive testing and brain MRI required for this analysis. This sample (N = 18) had a mean age of 73.8 years (SD = 6.1) and 15.6 years of education (SD = 2.5); self-reported sex assigned at birth was 50% female (n = 9) and race/ethnicity was predominantly White, Non-Hispanic/Latino (n = 17, 94%; one participant identified as Black/African American, Non-Hispanic/Latino).

### 2.2. Procedures

This open-label phase I trial was registered on ClinicalTrials.gov (NCT04503096) and approved by the Medical University of South Carolina Institutional Review Board (Pro00100536). The detailed trial procedures and primary outcomes (i.e., a-iTBS safety, feasibility, tolerability, and effects on cognition) have been reported previously [4]. Participants received 3 days of a-iTBS treatment and completed neurocognitive testing, neuropsychiatric assessments, and brain MRI within one week pre- and post-treatment. Data from the 4-week post-treatment assessment are not reported in the present study as it did not include the primary cognitive outcome measure or MRI. The privacy rights of human subjects have been observed and informed consent was obtained for experimentation with human subjects.

#### Accelerated iTBS Treatment.

**Fig. 1.**
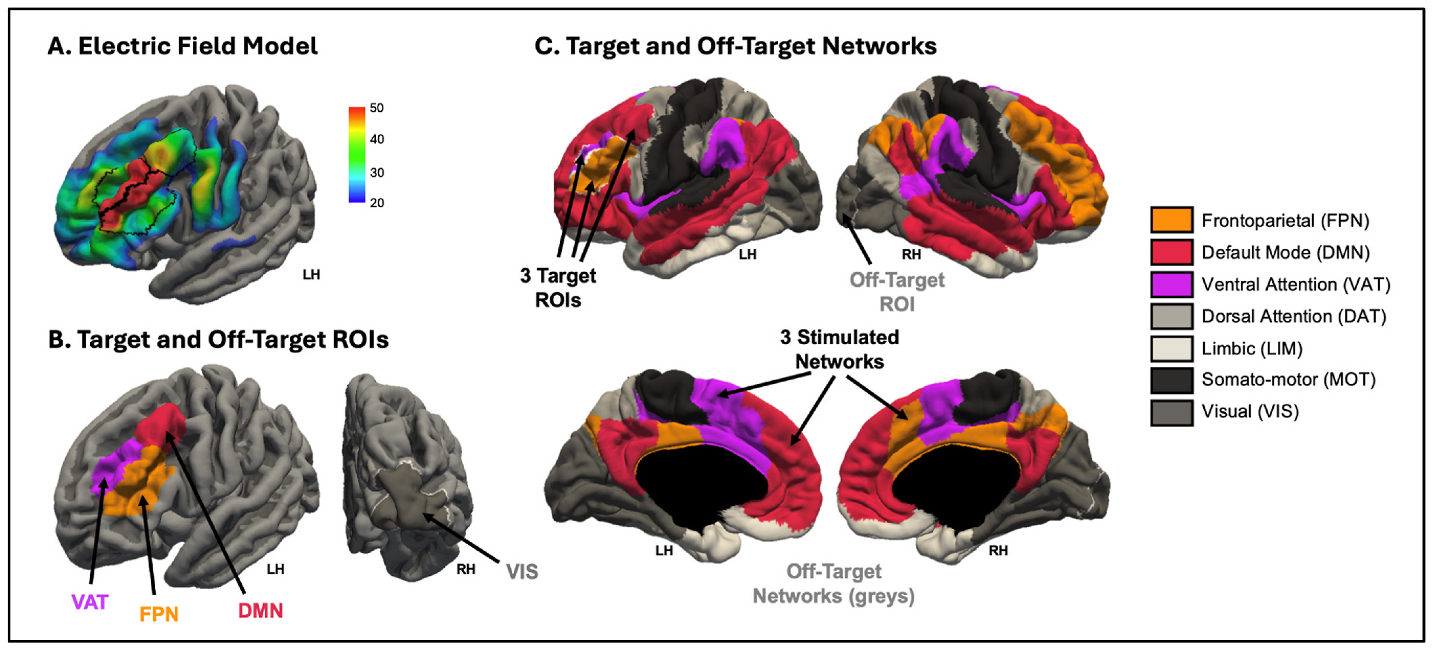
A. Electric field (e-field) model of cortical stimulation from iTBS to the F3 target in left dorsolateral prefrontal cortex (l-dlPFC) on fsaverage template brain with superimposed black outlines of the 3 selected target ROIs. B. The 3 target ROIs falling within the target l-dlPFC area, colored and labeled by network membership, and off-target ROI (grey). C. The 3 target networks (colored) containing the 3 target ROIs (outlined in white); the off-target networks and off-target ROI in greyscale.

Participants received a-iTBS on 3 days within an 8-day span consisting of 24 total stimulation sessions (8 sessions/day, approximately 2 hours per day). Treatment was delivered using a MagVenture R30 MagPro TMS System with Cool-B65 figure-8. The l-dlPFC was targeted by placing the coil at the Beam F3 location of the International 10–20 EEG system [19] (Fig. 1A). A Brainsight Neuronavigation system (version 2.4.8) ensured accuracy, reliability, and reproducibility of coil placement both within and between sessions [20]. Each session consisted of 600 pulses (14,400 total pulses): 50 Hz iTBS triplets delivered at a 5 Hz carrying frequency in 2 s trains repeated every 10 s (8 s inter-train interval) for 190 s at 120% of the resting motor threshold (rMT; *M* = 42.7%, *SD* = 5.3%, range = 35–55% maximal stimulator output). Inter-session intervals were 10–15 min or more accounting for participant comfort (in this study’s sample *M* = 10.7 min, *SD* = 1.2 min, range = 9–19 min). Participants engaged in computerized cognitive training (via the BrainHQ platform) during inter-session intervals; this was conducted to assess procedural feasibility, was not dosed for efficacy, and did not impact treatment outcomes [4].

### 2.3. Measures

#### Neurocognitive Testing.

Cognition was measured at pre- and post-treatment using the iPad-administered NIH Toolbox Cognition Battery (NIHTB-CB, version 2), which is well-suited for measuring change as the tests were designed to minimize floor and ceiling effects [21]. Participants completed four NIHTB-CB tests: flanker inhibitory control, list sorting working memory, pattern comparison processing speed, and dimensional change card sort. The picture sequence memory test was not administered due to its relatively large practice effect [21], thus mitigating potential inflation of post-treatment outcomes. Fluid cognition composite scores [22] were calculated by averaging the demographically adjusted (age, education, sex, race/ethnicity [23]) T-scores for the four NIHTB-CB tests. Higher fluid cognition T-scores (Normative *M* = 50, *SD* = 10) indicate better cognitive functioning.

#### Brain MRI: Acquisition.

MRI scans were collected on a 3T Prisma MRI system using a 32-channel head coil (Siemens Medical Solutions, Erlangen, Germany). The following sequences were acquired: (1) T1-weighted 3D imaging using an MPRAGE sequence with these parameters: TR/TI/TE = 2300/900/2.26 ms, FOV = 256 × 256 mm^2^, a generalized auto calibrating partially parallel acquisition (GRAPPA) factor of 2, voxel size 1.0 × 1.0 × 1.0 mm^3^; (2) 3 resting-state fMRI (rs-fMRI) sequences were acquired (240 vol each, 720 vol total) using echo-planar imaging (EPI) sequences with these parameters: TR/TE = 2000/36.0 ms, FOV = 94 × 94 × 69 mm, flip angle = 80^◦^, multi-band acceleration factor of 3, 69 interleaved axial slices, slice thickness = 2.2 mm, voxel size 2.2 × 2.2 × 2.2 mm^3^. A total of 24 min of rs-fMRI data were collected per participant.

#### Brain MRI: Preprocessing.

rs-fMRI data were preprocessed according to a previously published pipeline [24]. The steps were 1) discarding the first 4 vol, 2) slice timing correction using SPM2, 3) rigid-body head motion correction using FSL, 4) normalization for global mean signal intensity, 5) bandpass temporal filtering (0.01–0.08 Hz), and 6) regression of spurious variance and their derivatives, including head motion parameters, mean signal from whole-brain, white matter, and ventricular cerebrospinal fluid. Volumes were not censored based on head motion. Of the 19 participants with rs-fMRI data, one exhibited excessive head motion in both rs-fMRI runs, defined as mean framewise displacement (FD) > 0.30 and > 20% of volumes with FD > 0.50, and was excluded from analyses. For the remaining 18 participants, mean FD across runs was 0.26 (SD = 0.08). For the T1-weighted structural images, processing included 1) intensity normalization and 2) reconstruction of surface mesh representations of the cortex that were registered to a common spherical coordinate system.

#### Functional Connectivity Metrics.

**Fig. 2.**
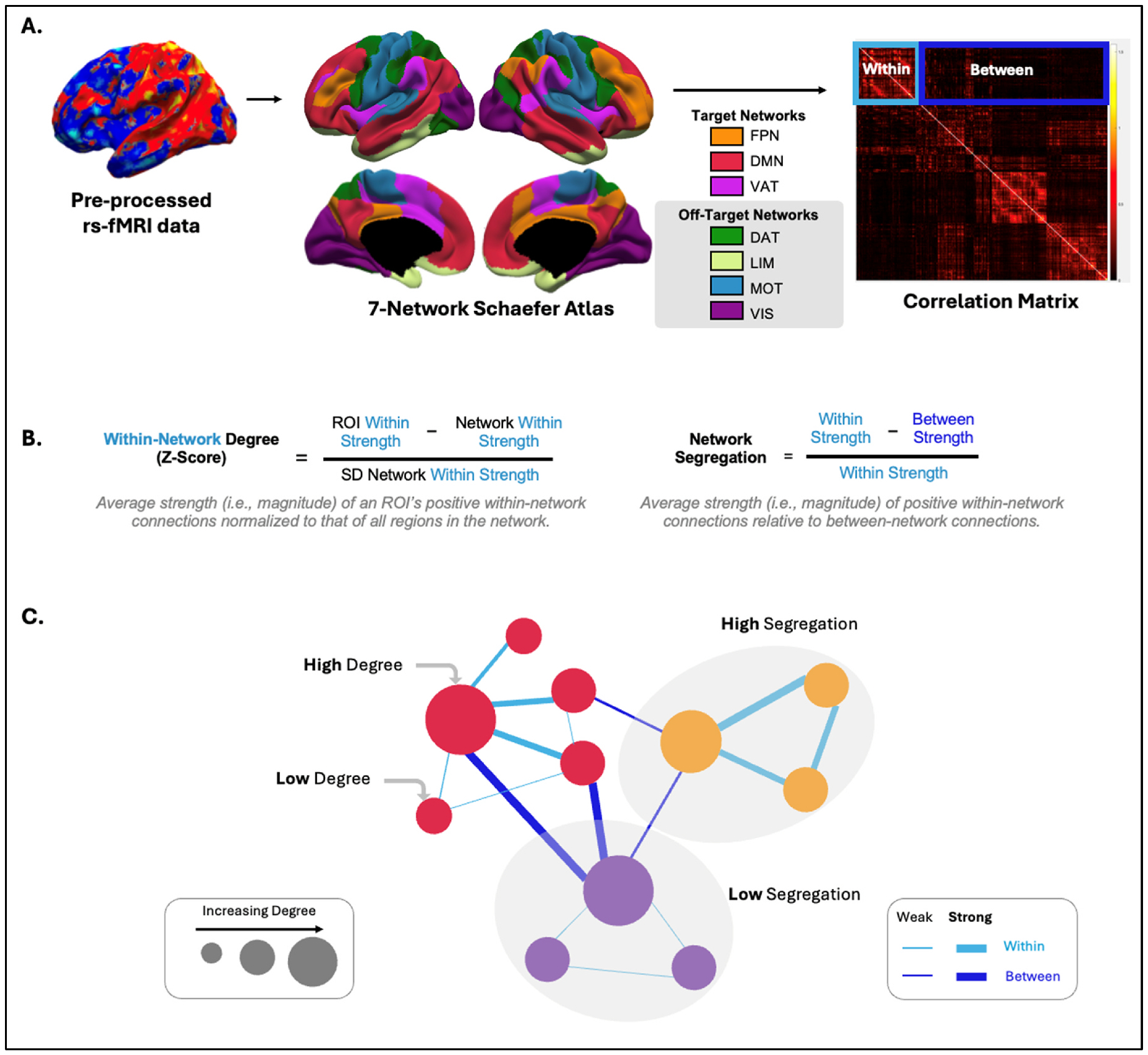
A. Overview of Processing Pipeline. Pre-processed rs-fMRI data were used to determine time courses for the 100 ROIs (i.e., nodes) of the 7-network Schaefer atlas. Time courses were correlated between each node pair, generating a correlation matrix (node X node) for each participant. Within-network connections are those between nodes belonging to the same network (light-blue square); between-network connections are those between nodes belonging to different networks (dark-blue square). B. Calculation of functional connectivity metrics (ROI-level: within-network degree z-score; network-level: segregation). C. Abstract representation of each metric showing networks (colors: red, orange, purple) with nodes (circles: larger indicates higher degree) and their functional connections (lines: light blue = within-network connections, dark blue = between-network connections, thicker lines = stronger connections). The example high degree ROI has stronger within-network connections than the low degree ROI. The example high segregation network has stronger average within-network relative to between-network connections; the opposite pattern is present for the low segregation network.

rs-fMRI volumes were registered with FreeSurfer v7.4.1 [25] to the T1-weighted structural images and the template fsaverage6 and converted into surface-based images. Next, using MATLAB R2023a, timeseries data for each vertex was extracted and averaged by node (i.e., region) using the 100-parcel Schaefer parcellation [26], which are clustered according to the 7 networks from the Yeo atlas [27]: frontoparietal (FPN), default mode (DMN), ventral attention (VAT), dorsal attention (DAT), limbic (LIM), somatomotor (MOT), and visual (VIS) (Fig. 2A). Functional connectivity was calculated as the Pearson correlation (*r*) between the time courses of each node pair, resulting in a 100-node × 100-node fully connected matrix for each participant. Correlation coefficients were normalized within-participant using Fisher’s *r*-to-*z* transformation. Only positive correlation coefficients were retained in the matrix, as is standard for calculating the connectivity metrics described below.

We *a priori* selected 3 target regions of interest (ROIs) from the left dorsolateral prefrontal cortex (l-dlPFC) as locations underlying the TMS coil positioned at F3 (Fig. 1A and B). We provide *post hoc* support for this selection using electric field (e-field) modeling (analysis described in Supplementary Material). These analyses showed that compared to other left prefrontal regions, the 3 target ROIs had higher group-average e-field magnitudes (Figs. S1A and S1B). These 3 target ROIs each belonged to a different functional network—FPN, DMN, and VAT—-which we then considered the 3 target networks (Fig. 1B and C). To serve as negative controls in our analyses, we selected an off-target ROI that is anatomically far from the stimulation site and not part of the targeted functional networks (i.e., right hemisphere primary visual; V1) and considered the remaining functional networks (i.e., DAT, LIM, MOT, and VIS) the off-target networks.

We next calculated functional connectivity metrics to capture local effects (i.e., within-network degree) and network-level effects (i.e., segregation) of a-iTBS (Fig. 2B). The Brain Connectivity MATLAB toolbox (version 2019–03-03 [28]) was used to calculate within-network degree z-score for the 3 target ROIs and the 1 off-target ROI using the module_degree_zscore.m function for an weighted, undirected graph masked to include only positive correlation values (code available online: brain-connectivity-toolbox.net). The within-network degree z-score of an ROI is calculated as:

$$Z_{ROI}=\frac{\mu_{ROI}-\mu_{\text{network}}}{\sigma_{\text{network}}}$$

where μ is the mean strength of positive within-network connections (i. e., magnitude of positive correlations) calculated for the target region (i.e., *μ*_*ROI*_) and all ROIs in the network (i.e., *μ*_*network*_), and *σ*_*network*_ is the standard deviation of the strength of positive within-network connections, which is calculated for all ROIs in the network. Higher within-network degree z-scores indicate that an ROI has stronger within-network connectivity relative to other regions in the network, and hence is acting as a “hub” in its network [29]. Network segregation was calculated as is standard in the literature [30]:

$$\text{Network Segregation}=\frac{\mu_{\text{within}}-\mu_{\text{between}}}{\mu_{\text{within}}}$$

where *μ*_*within*_ is the mean strength of positive connections (i.e., magnitude of positive correlations) calculated for each pair of ROIs belonging to the network of interest and *μ*_*between*_ is the mean strength of positive connections between ROIs of the network of interest and ROIs of all other networks. Higher segregation values indicate that the network is more strongly connected within itself than to other networks [15]. For target networks, we calculated segregation *from* the other target networks, meaning strength of connectivity within the network of interest vs. between it and the other 2 target networks (e.g., within FPN relative to between FPN-DMN and FPN-VAT). This was calculated for each of the 3 target networks separately and as an aggregate metric of the 3 target networks. For off-target networks, we calculated segregation *from* other off-target networks in this same way (considering only off-target networks: DAT, LIM, VIS, MOT).

### 2.4. Statistical analyses

Pre-post change scores (i.e., post-minus pre-treatment) were computed for fluid cognition and each functional connectivity metric (i. e., within-network degree z-score of each of the 3 target ROIs and the off-target V1; segregation of each of the 3 target networks and the off-target networks). Each variable was assessed for normality using Shapiro-Wilk tests; all were found to be normally distributed (*p*-values >0.14). None of the change scores were found to be statistical outliers (defined as >±2.5 SD from the sample’s mean). The associations between change in each functional connectivity metric and change in cognition were evaluated using Pearson’s correlations. Follow-up sensitivity analyses were conducted via linear models predicting change in cognition from the connectivity metric of interest while covarying demographic characteristics (i.e., age, sex, and education) and the pre-treatment connectivity metric. Pre-to post-treatment changes in fluid cognition and connectivity metrics were evaluated using paired samples *t*-tests; effect sizes were computed using Cohen’s *d*. Given the preliminary nature of this Phase I trial with a modest sample size, we focused our interpretations on effect sizes as these are most informative for guiding future studies.

## 3. Results

### 3.1. ROI within-network degree

**Fig. 3.**
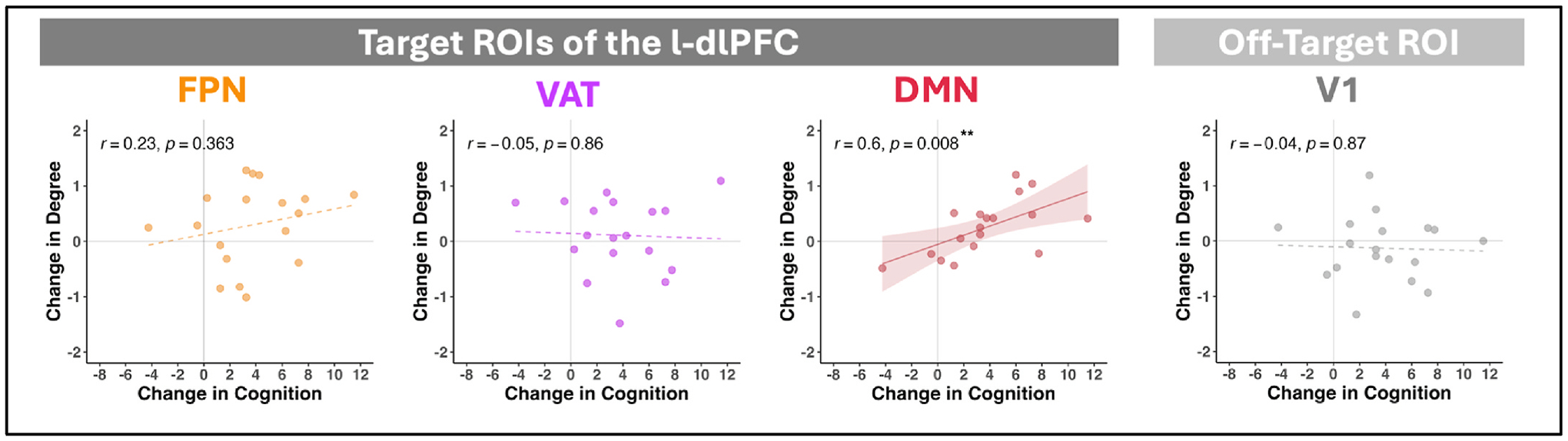
Associations between iTBS-related changes in local connectivity and cognition. For each of the 3 target ROIs of the l-dlPFC (left 3 plots) and the off-target ROI (right plot), scatterplots show the relationship between change in within-network degree z-score (y-axis) and change in fluid cognition T-scores (x-axis). Points represent individual participants. Pearson correlation coefficients and *p*-values are presented for each. Significant associations are indicated by solid trendlines and a shaded 95 % confidence interval band; non-significant associations have dotted trendlines. Notes. **p < .01. FPN: frontoparietal network, VAT: ventral attention network, DMN: default mode network, V1: right hemisphere primary visual.

We replicated the significant large effect-size improvement in fluid cognition reported in the Phase I trial (N = 21; [4]) in this sub-sample of 18 participants with usable MRI data [*t*(17) = −4.19, *p* < .001, *d* = 0.99]. We then evaluated whether change in local connectivity (i.e., within-network degree) from pre-to-post treatment for each of the 3 target ROIs (Fig. 1) was associated with change in cognition (Fig. 3). There was a strong, large effect size and significant association between increased within-network degree and improved cognition for the target DMN ROI (*r* = 0.60, *p* = .008). This effect was not observed for the other two target ROIs (FPN: *r* = 0.23, *p* = .36; VAT: *r* = −0.05, *p* = .86). In the off-target V1 ROI (i.e., negative-control), there was a non-significant and near-zero correlation between change in within-network degree and change in cognition (*r* = −0.04, *p* = .87).

### 3.2. Network segregation

**Fig. 4.**
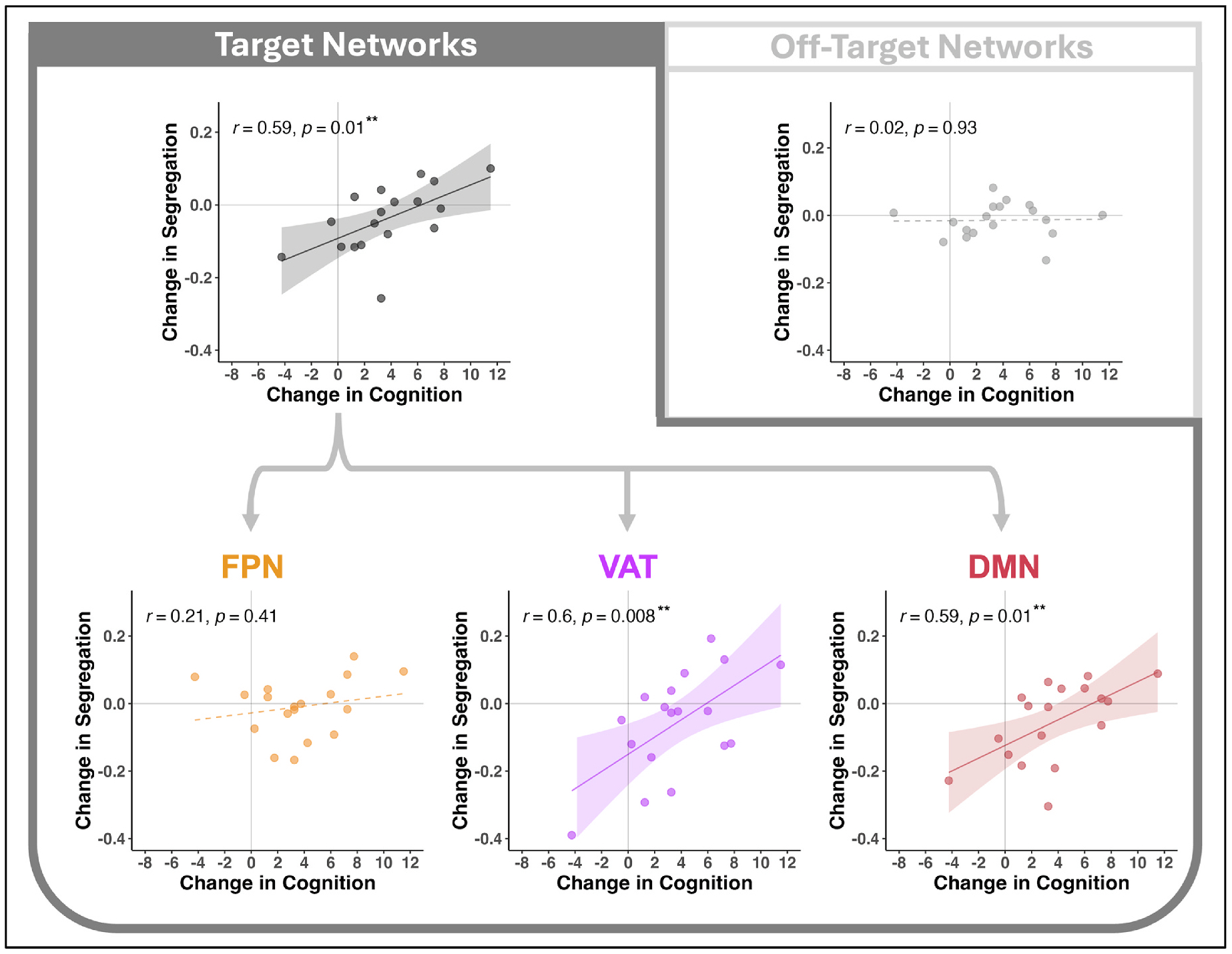
Associations between iTBS-related changes in network-level connectivity and cognition. Scatterplots show the relationships between change in network segregation (y-axis) and change in fluid cognition T-scores (x-axis). Results are presented for average segregation of all 3 target networks (top left), each target network separately (bottom row), and for average segregation of off-target networks (top right). Points represent individual participants. Pearson correlation coefficients and *p*-values are presented for each. Significant associations are indicated by solid trendlines and a shaded 95 % confidence interval band; non-significant associations have dotted trendlines. **p < .01.

We next tested whether change in segregation from pre-to-post treatment for each of the 3 target networks (Fig. 1) was associated with change in fluid cognition (Fig. 4). Increased network segregation was significantly related to improved cognition from pre-to post-treatment for the aggregate metric of segregation across all 3 target networks, with a large effect size (*r* = 0.59, *p* = .009). For the 3 individual target networks, the effect was also large in magnitude for VAT (*r* = 0.60, *p* = .008) and DMN (*r* = 0.59, *p* = .01), and directionally consistent but not significant (small effect size) for FPN (*r* = 0.21, *p* = .41). For the negative-control metric of segregation amongst off-target networks, there was no significant association between change in network segregation and change in cognition (*r* = 0.02, *p* = .93).

### 3.3. Sensitivity analyses

There was considerable variability in baseline connectivity metrics and the direction and magnitude of change in connectivity (SDs reported in Table 1, range visualized in Figs. 3 and 4), which may be expected given that people tend to vary in their response to treatments such as rTMS. As such, since the primary goal of the parent Phase I trial was to first determine safety of accelerated iTBS in an MCI population, it was insufficiently powered to detect group-level changes in connectivity following a-iTBS treatment. Nonetheless, for completeness, we report preliminary change results for each connectivity metric in Table 1; the only effect reaching statistical significance was a medium effect size increase in DMN ROI within-network degree. To account for measured sources of variability, we conducted sensitivity analyses for each effect reported above by including demographic factors (i.e., age, sex, education), pre-treatment fluid cognition, and the relevant pre-treatment connectivity metric. None of the covariates had significant effects in the models, and we found no change in the pattern of results. That is, improvement in cognition remained significantly related to increased within-network degree of the target DMN ROI (*B* = 6.02, SE = 2.75, *p* = .05) and increased segregation of the target networks (*B* = 24.82, SE = 8.70, *p* = .02), including significant effects for DMN (*B* = 20.28, SE = 7.27, *p* = .02) and VAT (*B* = 13.37, SE = 5.74, *p* = .04) but not the FPN (*B* = 15.43, SE = 11.90, *p* = .22). We also found that average e-field magnitude, calculated at the F3 ROI for each participant as described previously [4] (*M* = 63.96 V/m, *SD* = 9.17; range = 48.53–82.83 V/m), was not significantly correlated with change in any of the connectivity metrics: within-network degree of target ROIs (FPN: *r* = 0.34, *p* = .17; DMN: *r* = 0.32, *p* = .20; VAT: *r* = 0.17, *p* = .49) or segregation of target networks (Overall: *r* = 0.16, *p* = .52; FPN: *r* = −0.28, *p* = .26; DMN: *r* = 0.05, *p* = .85; VAT: *r* = 0.11, *p* = .67)

## 4. Discussion

This study sought to further demonstrate target engagement of a-iTBS to l-dlPFC in people with MCI by evaluating whether treatment produces changes in local and network-level functional connectivity that relate to change in cognition. We provide compelling evidence of target engagement, demonstrating that iTBS-related changes in connectivity of stimulated target functional networks, and to a lesser extent target regions, relates to the magnitude of cognitive improvement. Critically, we show that these effects were not present in negative-control regions and networks (i.e., those expected to be least influenced by a-iTBS), increasing our confidence that the observed improvements in cognition are attributable to treatment despite this being an open-label trial.

Consistent with our hypotheses, we found that improved cognition was associated with increases in within-network degree for target ROIs and segregation for target networks. At the local level, the extent to which the target DMN ROI became more extensively connected to other regions (indicated by increased within-network degree) following treatment related to greater improvement in cognition. We also observed a significant increase in within-network degree of the target DMN ROI (medium effect size), suggesting that a-iTBS treatment may cause stimulated regions to become more hub-like within their respective network, a feature generally associated with better cognition [31]. The other two target ROIs (FPN and VAT) also demonstrated numeric increases in degree, but these were not significant, warranting investigation in future studies powered to detect these smaller effects if they exist. At the network-level, there were no significant changes in network segregation metrics, but we observed strong associations (medium to large effect sizes) between increased segregation of target networks and improved cognition. Therefore, although there was not a consistent change in segregation at the group level (likely related to the variability within the sample and limited power), individual differences in the extent to which segregation increased related to greater cognitive improvement. Functional network segregation decreases with aging (i. e., networks become less differentiated from each other) [30], a pattern that is further exacerbated in the context of MCI and AD and related to the severity of cognitive deficits [32]. Thus, our findings suggest that a-iTBS treatment may improve dysfunctional network interactions in MCI by increasing the extent to which stimulated networks are functionally differentiated (i.e., more connected within themselves relative to other networks), thus enhancing cognition.

These network-level effects are likely subserved by multiple neuromodulatory mechanisms (e.g., changes in neurotransmitters, cellular connections, structural connectivity, cortical inhibition and facilitation, etc.), which are reviewed elsewhere [33]. Most relevant to this analysis are theories suggesting that distributed rTMS effects may be driven by stimulation of local cortex causing widescale reorganization of functionally connected regions [6]. That is, local stimulation may have a downstream effect on the activity/neural synchrony in connected regions and networks as the brain attempts to maintain functional homeostasis. Consistent with this, we found that the extent to which local stimulation of l-dlPFC regions (belonging to FPN, DMN, and VAT networks) impacts connectivity of these networks relates to greater cognitive enhancement; an effect not observed for the off-target (i.e., negative-control) networks. Although we focused our analysis on target vs. off-target networks as a parsimonious way to maximize our power to generate preliminary effect estimates, future work should investigate network-level changes with more granularity (e.g., all 7 networks separately) given that changes in connectivity following rTMS have previously been observed outside of stimulated networks [6]. Our observation of less consistent and weaker effects at the local (i.e., region) level may reflect changes in rTMS effects over time, with some evidence showing that immediate local changes dissipate and progress to network-level effects over the course of an hour [34,35]. Another potential mechanism to consider is propagation of local stimulation to anatomically connected regions along white matter tracts [36], which is particularly relevant to MCI/AD given the impacts of neurodegeneration on both functional and structural connectivity [37]. Using white matter tracts to guide investigation at the ROI level and considering structural features (e.g., white matter tract integrity) as a treatment moderator in larger trials is an important future direction.

This Phase I trial has important limitations, which we discussed previously [4], related to trial design (single-arm, open-label) and sample selection (syndromic vs. biomarker-based MCI definition), small sample size, and representativeness. Of particular relevance to this study’s goal of evaluating target engagement, the lack of randomization to sham condition (i.e., a control group) limits the causal inferences we can make regarding the effects of a-iTBS treatment. To contend with this, we included negative-controls which allowed us to demonstrate that the observed effects were specific to regions and networks expected to be impacted by l-dlPFC a-iTBS. Additionally, we are collecting fMRI data in our ongoing Phase II randomized, sham-controlled, dose-ranging trial (NCT05992831) with which to replicate this preliminary evidence of target engagement with additional treatment controls in a larger sample. Subsequent larger studies may extend our preliminary findings by answering questions that this study was insufficiently powered to address such as potential differences in connectivity change between treatment responders and non-responders (e.g., change in responders only or opposite direction effects in these groups [6]) and further examining functional connectivity changes at multiple levels of specificity (e.g., voxel-wise, regional, within-network such as local efficiency, and global such as modularity). Lastly, we chose fluid cognition as the outcome measure since it is a composite of tests of higher-order cognitive functions that are expected to be impacted by l-dlPFC stimulation. We selected this composite in the interest of psychometric rigor, but this precluded analyses of specific cognitive domains that may have been preferentially improved by a-iTBS. Future work should therefore further evaluate these effects using psychometrically robust measures of discrete cognitive domains, such as episodic memory, that are feasibly administered in clinical trial contexts.

## 5. Conclusions

This study provides novel evidence of target engagement for a-iTBS to l-dlPFC in people with MCI by describing treatment-related changes in brain functional connectivity that relate to significant cognitive improvements. This effect was most robust at the network level, suggesting that a-iTBS may improve dysfunctional network interactions in MCI, thereby enhancing cognitive function. These findings provide important insights into the potential neural mechanisms of iTBS-induced improvements in cognition and further underscore the potential of a-iTBS as an effective treatment for MCI.

## Supplementary Material

1

## Figures and Tables

**Table 1:** Descriptive statistics and tests of change from pre-to post-treatment in cognition and functional connectivity.

	Pre-Treatment Assessment		Post-Treatment Assessment		Change (Post - Pre)		Tests of Change Over Time		
	Mean	SD	Mean	SD	Mean	SD	*t*-stat	p-value	Cohen’s d
**Cognition**									
NIHTB-CB Fluid Cognition Composite (T-score)	39.17	7.79	42.81	7.93	3.68	3.63	−4.19	**<0.001**	**0.99**
**Functional Connectivity**									
Within-Network Degree (Z-score)									
Target FPN ROI	−0.75	0.59	−0.45	0.60	0.29	0.73	1.71	0.106	0.4
Target DMN ROI	0.03	0.95	0.28	0.71	0.25	0.50	2.12	**0.049**	**0.5**
Target VAT ROI	−0.84	0.73	−0.73	0.44	0.11	0.68	0.69	0.501	0.16
Off-Target V1 ROI	−0.37	0.68	−0.50	0.79	−0.13	0.59	−0.94	0.359	0.22
Network Segregation									
Target FPN	0.47	0.09	0.46	0.12	−0.01	0.09	−0.46	0.652	0.11
Target DMN	0.61	0.12	0.55	0.13	−0.05	0.12	−1.97	0.066	0.46
Target VAT	0.67	0.19	0.61	0.25	−0.06	0.15	−1.55	0.138	0.37
Target Networks	0.60	0.12	0.56	0.13	−0.04	0.09	−1.77	0.094	0.42
Off-Target Networks	0.78	0.04	0.77	0.05	−0.01	0.05	−1.21	0.243	0.29

Notes. NIHTB-CB: National Institutes of Health Toolbox Cognition Battery; ROI: Region of Interest; FPN: Frontoparietal Network; DMN: Default Mode Network; VAT: Ventral Attention Network; V1: Primary Visual.

## References

[1] Zhang X, Lan X, Chen C, Ren H, Guo Y. Effects of repetitive transcranial magnetic stimulation in patients with mild cognitive impairment: a meta-analysis of randomized controlled trials. Front Hum Neurosci 2021;15:723715. 10.3389/fnhum.2021.723715. 34764859 PMC8576192

[2] Jiang L, Cui H, Zhang C, Cao X, Gu N, Zhu Y, Repetitive transcranial magnetic stimulation for improving cognitive function in patients with mild cognitive impairment: a systematic review. Front Aging Neurosci 2021;12:477. 10.3389/fnagi.2020.593000 PMC7842279 33519418

[3] Sharbafshaaer M, Gigi I, Lavorgna L, Esposito S, Bonavita S, Tedeschi G, Repetitive transcranial magnetic stimulation (rTMS) in mild cognitive impairment: effects on cognitive functions—a systematic review. J Clin Med 2023;12:6190. 37834834 10.3390/jcm12196190 PMC10573645

[4] Aghamoosa S, Lopez J, Rbeiz K, Fleischmann HH, Horn O, Madden K, A phase I trial of accelerated intermittent theta burst rTMS for amnestic MCI. J Neurol Neurosurg Psychiatry 2024. 10.1136/jnnp-2023-332680. jnnp-2023–332680. PMC11483208 38719432

[5] Fox MD, Buckner RL, Liu H, Chakravarty MM, Lozano AM, Pascual-Leone A. Resting-state networks link invasive and noninvasive brain stimulation across diverse psychiatric and neurological diseases. Proc Natl Acad Sci 2014;111: E4367–75. 10.1073/pnas.1405003111. 25267639 PMC4205651

[6] Beynel L, Powers JP, Appelbaum LG. Effects of repetitive transcranial magnetic stimulation on resting-state connectivity: a systematic review. Neuroimage 2020; 211:116596. 10.1016/j.neuroimage.2020.116596. 32014552 PMC7571509

[7] Menon V, D’Esposito M. The role of PFC networks in cognitive control and executive function. Neuropsychopharmacol Off Publ Am Coll Neuropsychopharmacol 2022;47:90–103. 10.1038/s41386-021-01152-w. PMC8616903 34408276

[8] Guarino A, Forte G, Giovannoli J, Casagrande M. Executive functions in the elderly with mild cognitive impairment: a systematic review on motor and cognitive inhibition, conflict control and cognitive flexibility. Aging Ment Health 2020;24: 1028–45. 10.1080/13607863.2019.1584785. 30938193

[9] Buckner RL. Memory and executive function in aging and AD: multiple factors that cause decline and reserve factors that compensate. Neuron 2004;44:195–208. 10.1016/j.neuron.2004.09.006. 15450170

[10] Chang Y-L, Jacobson MW, Fennema-Notestine C, Hagler DJ, Jennings RG, Dale AM, Level of executive function influences verbal memory in amnestic mild cognitive impairment and predicts prefrontal and posterior cingulate thickness. Cereb Cortex 2010;20:1305–13. 10.1093/cercor/bhp192. N Y N 1991. 19776343 PMC2912652

[11] Anderson ND, Ebert PL, Jennings JM, Grady CL, Cabeza R, Graham SJ. Recollection- and familiarity-based memory in healthy aging and amnestic mild cognitive impairment. Neuropsychology 2008;22:177–87. 10.1037/0894-4105.22.2.177. 18331160

[12] Badhwar A, Tam A, Dansereau C, Orban P, Hoffstaedter F, Bellec P. Resting-state network dysfunction in Alzheimer’s disease: a systematic review and meta-analysis. Alzheimers Dement Diagn Assess Dis Monit 2017;8:73–85. 10.1016/j.dadm.2017.03.007 PMC5436069 28560308

[13] Sullivan MD, Anderson JAE, Turner GR, Spreng RN. Intrinsic neurocognitive network connectivity differences between normal aging and mild cognitive impairment are associated with cognitive status and age. Neurobiol Aging 2019;73: 219–28. 10.1016/j.neurobiolaging.2018.10.001. 30391818 PMC6251760

[14] Ewers M, Luan Y, Frontzkowski L, Neitzel J, Rubinski A, Dichgans M, Segregation of functional networks is associated with cognitive resilience in Alzheimer’s disease. Brain 2021;144:2176–85. 10.1093/brain/awab112. 33725114 PMC8370409

[15] Wig GS. Segregated systems of human brain networks. Trends Cogn Sci 2017;21: 981–96. 10.1016/j.tics.2017.09.006. 29100737

[16] Albert MS, DeKosky ST, Dickson D, Dubois B, Feldman HH, Fox NC, The diagnosis of mild cognitive impairment due to Alzheimer’s disease: recommendations from the National Institute on Aging-Alzheimer’s Association workgroups on diagnostic guidelines for Alzheimer’s disease. Alzheimers Dement 2011;7:270–9. 10.1016/j.jalz.2011.03.008. 21514249 PMC3312027

[17] Bondi MW, Edmonds EC, Jak AJ, Clark LR, Delano-Wood L, McDonald CR, Neuropsychological criteria for mild cognitive impairment improves diagnostic precision, biomarker associations, and progression rates. J Alzheimers Dis JAD 2014;42:275–89. 10.3233/JAD-140276. 24844687 PMC4133291

[18] Fountain-Zaragoza S, Braun SE, Horner MD, Benitez A. Comparison of conventional and actuarial neuropsychological criteria for mild cognitive impairment in a clinical setting. J Clin Exp Neuropsychol 2021;43:753–65. 10.1080/13803395.2021.2007857. 34962226 PMC8881966

[19] Beam W, Borckardt JJ, Reeves ST, George MS. An efficient and accurate new method for locating the F3 position for prefrontal TMS applications. Brain Stimul 2009;2:50–4. 10.1016/j.brs.2008.09.006. 20539835 PMC2882797

[20] Caulfield KA, Fleischmann HH, Cox CE, Wolf JP, George MS, McTeague LM. Neuronavigation maximizes accuracy and precision in TMS positioning: evidence from 11,230 distance, angle, and electric field modeling measurements. Brain Stimul 2022;15:1192–205. 10.1016/j.brs.2022.08.013. 36031059 PMC10026380

[21] Weintraub S, Dikmen SS, Heaton RK, Tulsky DS, Zelazo PD, Slotkin J, The cognition Battery of the NIH toolbox for assessment of neurological and behavioral function: validation in an adult sample. J Int Neuropsychol Soc JINS 2014;20: 567–78. 10.1017/S1355617714000320. 24959840 PMC4103959

[22] Heaton RK, Akshoomoff N, Tulsky D, Mungas D, Weintraub S, Dikmen S, Reliability and validity of composite scores from the NIH toolbox cognition Battery in adults. J Int Neuropsychol Soc 2014;20:588–98. 10.1017/S1355617714000241. 24960398 PMC4103963

[23] Casaletto KB, Umlauf A, Beaumont J, Gershon R, Slotkin J, Akshoomoff N, Demographically corrected normative standards for the English version of the NIH toolbox cognition Battery. J Int Neuropsychol Soc JINS 2015;21:378–91. 10.1017/S1355617715000351. 26030001 PMC4490030

[24] Wang D, Buckner RL, Fox MD, Holt DJ, Holmes AJ, Stoecklein S, Parcellating cortical functional networks in individuals. Nat Neurosci 2015;18:1853–60. 10.1038/nn.4164. 26551545 PMC4661084

[25] Fischl B FreeSurfer. Neuroimage 2012;62:774–81. 10.1016/j.neuroimage.2012.01.021. 22248573 PMC3685476

[26] Schaefer A, Kong R, Gordon EM, Laumann TO, Zuo X-N, Holmes AJ, Local-global parcellation of the human cerebral cortex from intrinsic functional connectivity MRI. Cereb Cortex N Y N 1991 2018;28:3095–114. 10.1093/cercor/bhx179. PMC6095216 28981612

[27] Thomas Yeo BT, Krienen FM, Sepulcre J, Sabuncu MR, Lashkari D, Hollinshead M, The organization of the human cerebral cortex estimated by intrinsic functional connectivity. J Neurophysiol 2011;106:1125–65. 10.1152/jn.00338.2011. 21653723 PMC3174820

[28] Rubinov M, Kötter R, Hagmann P, Sporns O. Brain connectivity toolbox: a collection of complex network measurements and brain connectivity datasets. Neuroimage 2009;47:S169. 10.1016/S1053-8119(09)71822-1.

[29] Power JD, Schlaggar BL, Lessov-Schlaggar CN, Petersen SE. Evidence for hubs in human functional brain networks. Neuron 2013;79:798–813. 10.1016/j.neuron.2013.07.035. 23972601 PMC3838673

[30] Chan MY, Park DC, Savalia NK, Petersen SE, Wig GS. Decreased segregation of brain systems across the healthy adult lifespan. Proc Natl Acad Sci U S A 2014;111: E4997–5006. 10.1073/pnas.1415122111. 25368199 PMC4246293

[31] Bertolero MA, Yeo BTT, Bassett DS, D’Esposito M. A mechanistic model of connector hubs, modularity and cognition. Nat Hum Behav 2018;2:765–77. 10.1038/s41562-018-0420-6. 30631825 PMC6322416

[32] Meeker KL, Ances BM, Gordon BA, Morris JC, Benzinger TLS, Waring J. Default mode network dedifferentiation predicts cognitive performance in Alzheimer disease. Alzheimers Dement 2020;16:e044790. 10.1002/alz.044790.

[33] Fitzgerald PB, Daskalakis ZJ. The mechanism of action of rTMS. In: Fitzgerald PB, Daskalakis ZJ, editors. RTMS treat. Depress. Pract. Guide Cham: Springer International Publishing; 2022. p. 13–28. 10.1007/978-3-030-91519-3_3.

[34] Tang Y, Jiao X, Wang J, Zhu T, Zhou J, Qian Z, Dynamic functional connectivity within the fronto-limbic network induced by intermittent theta-burst stimulation: a pilot study. Front Neurosci 2019;13. 10.3389/fnins.2019.00944. 31572111 PMC6753168

[35] Battelli L, Grossman ED, Plow EB. Local immediate versus long-range delayed changes in functional connectivity following rTMS on the visual attention network. Brain Stimul 2017;10:263–9. 10.1016/j.brs.2016.10.009. 27838275 PMC5316353

[36] Vink JJT, Mandija S, Petrov PI, van den Berg CAT, Sommer IEC, Neggers SFW. A novel concurrent TMS-fMRI method to reveal propagation patterns of prefrontal magnetic brain stimulation. Hum Brain Mapp 2018;39:4580–92. 10.1002/hbm.24307. 30156743 PMC6221049

[37] Filippi M, Basaia S, Canu E, Imperiale F, Magnani G, Falautano M, Changes in functional and structural brain connectome along the Alzheimer’s disease continuum. Mol Psychiatr 2020;25:230–9. 10.1038/s41380-018-0067-8 29743583

