# Supplementary material for: Accelerated iTBS-Induced changes in resting-state functional connectivity correspond with cognitive improvement in amnestic MCI
Source: Brain Stimul. Author manuscript; Available in PMC 2025 May 28. (PMC12118152; doi:10.1016/j.brs.2025.04.012)
Supplement: 1 [file NIHMS2084000-supplement-1.docx]

**Supplementary Material**

**Electric Field (E-Field) Modeling.**

We conducted electric field (E-field) modeling using SimNIBS v4.0.1 [1] to estimate the distribution of cortical stimulation on a standard template brain (fsaverage) and for each participant using their acquired T1w MRI scans, as described previously [2], based on a MagVenture_Cool-B65 coil positioned over F3 with 120% rMT stimulation intensity. Additional parameters included coil angle set to 45° to the sagittal plane, maximal dI/dt machine-coil output of 150e6 A/s (e.g., 50% of machine output simulated at 75e6 A/s), and default tissue conductivity values: Scalp (0.465 S/m), spongy bone (0.025 S/m), compact bone (0.008 S/m), cerebrospinal fluid (1.654 S/m), gray matter (0.275 S/m), white matter (0.126 S/m), eyeballs (0.50 S/m), blood vessels (0.60 S/m), and muscle (0.16 S/m).

**E-Field Modeling Support for ROI Selection**

Using the individual e-field models of F3 stimulation, registered to standard fsaverage space, we calculated group-average e-field magnitude (V/m). This analysis generated two outputs: 1) a surface file (in fsaverage space) containing estimated stimulation intensity at the brain’s surface and 2) a 3D volume file (in MNI space) containing estimated stimulation intensity within cortical tissue. We first visualized the outlines of the 3 target ROIs l-dlPFC belonging to the VAT, FPN, and DMN and adjacent left prefrontal cortex (l-PFC) ROIs from the 100-region Schaefer parcellation[3] on the group-average e-field magnitude surface in fsaverage space using FreeSurfer (Figure S1A). This confirmed that the 3 target ROIs indeed fell more directly within the central focus of stimulation than adjacent l-PFC ROIs. To provide *post hoc* support for our selection of the 3 target ROIs, we calculated group-average volumetric e-field magnitude in each of the 3 target ROIs and adjacent l-PFC ROIs from the 100-region Schaefer parcellation[3], visualized in fsaverage space using FreeSurfer (Figure S1B). This confirmed that the 3 target ROIs had numerically higher average e-field magnitudes than the adjacent l-PFC ROIs (Figure S1B Table).

**
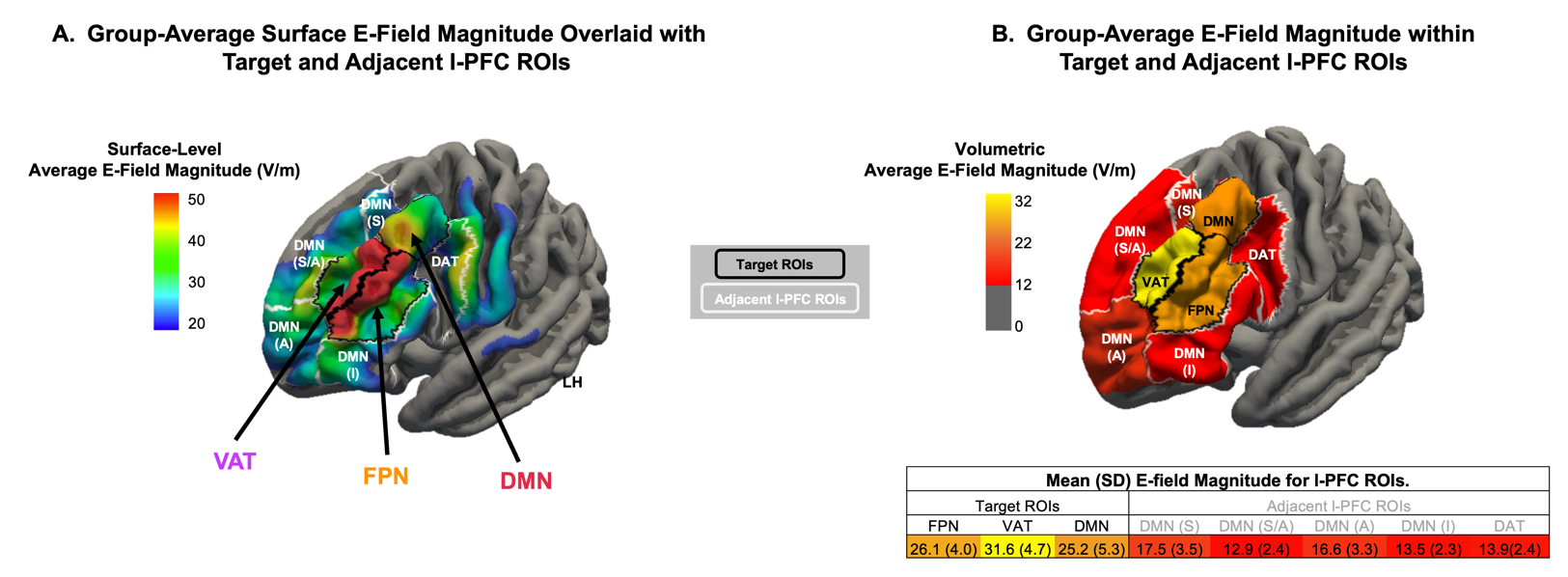
**

**Figure S1**. E-field Support for ROI Selection. A) Heatmap of surface-level group-average e-field magnitude of F3 stimulation on fsaverage brain, overlaid with the 3 target ROIs belonging to VAT, FPN, and DMN (black outlines) and adjacent l-PFC ROIs (white outlines). B) Group-average ROI-level (i.e., volumetric) e-field magnitude for target ROIs (black outlines) and adjacent l-PFC ROIs (white outlines) on fsaverage brain. The table reports mean and standard deviation (SD) e-field magnitude for each target ROI (VAT, FPN, DMN) and adjacent l-PFC ROI (DMN [S]: superior, DMN [S/A]: superior/anterior, DMN [A]: anterior, DMN [I]: inferior, DAT).

***Note.*** ROI: region of interest, e-field: electric field, l-PFC: left prefrontal cortex, VAT: ventral attention network, FPN: frontoparietal network, DMN: default mode network, DAT: dorsal attention network.

References

[1] Saturnino GB, Puonti O, Nielsen JD, Antonenko D, Madsen KH, Thielscher A. SimNIBS 2.1: A Comprehensive Pipeline for Individualized Electric Field Modelling for Transcranial Brain Stimulation. In: Makarov S, Horner M, Noetscher G, editors. Brain and Human Body Modeling: Computational Human Modeling at EMBC 2018, Cham: Springer International Publishing; 2019, p. 3–25. https://doi.org/10.1007/978-3-030-21293-3_1.

[2] Aghamoosa S, Lopez J, Rbeiz K, Fleischmann HH, Horn O, Madden K, et al. A phase I trial of accelerated intermittent theta burst rTMS for amnestic MCI. J Neurol Neurosurg Psychiatry 2024:jnnp-2023-332680. https://doi.org/10.1136/jnnp-2023-332680.

[3] Schaefer A, Kong R, Gordon EM, Laumann TO, Zuo X-N, Holmes AJ, et al. Local-Global Parcellation of the Human Cerebral Cortex from Intrinsic Functional Connectivity MRI. Cereb Cortex 2018;28:3095–114. https://doi.org/10.1093/cercor/bhx179.
